# How does geographical scale affect the effectiveness of public health heat-wave prevention plans?: the case of Spain

**DOI:** 10.1007/s00484-026-03158-9

**Published:** 2026-02-24

**Authors:** C. Linares, J. A. López-Bueno, M. A. Navas, M. S. Ascaso, S. González, Julio Díaz

**Affiliations:** 1 Department of Climate Change, Health and Urban Environment, National School of Health, Carlos III Institute of Health, Avda. Monforte de Lemos, 2, Madrid, Spain; 2 Subdirectorate-General for Environmental and Occupational Health, Ministry of Health, Madrid, Spain

## Abstract

The summer of 2025 has been the hottest in Spain since records began, 2022registering temperatures one tenth of a degree Celsius higher than the hottest on record to date, i.e., the summer of 2022. However, mortality attributable to heat wave temperatures in 2025 was 908 deaths fewer than in 2022. Of the many factors that may have influenced the reduction in attributable mortality in 2025 with respect to 2022, this study sought to analyse the possible impact of an improvement made to heat-wave prevention plans in the form of enhancing the geographical resolution of health alerts. Using the annual heat wave intensity registered and the annual mortality attributable to these heat waves, a linear adjustment between these two variables was performed for all Spanish provinces in the above two periods. The results indicated that whereas the increase in annual mortality for every one-degree rise in annual heat wave intensity (mean slope of the lines of fit for Spain as a whole) from 2015 to 2023 was 1.74 deaths/⁰C, in the period 2024–2025 it was 1.66 deaths/⁰C. This result translates as follows: if annual attributable mortality in Spain were calculated for 2025 using the values of the slopes for the first period, this would yield a total of 4,082 attributable deaths, but if this same attributable mortality were calculated using the values of the slope corresponding to the 2024–2025 period, this would yield a total of 3,894 deaths. In conclusion, although this analysis has not taken into account many factors that could relate heat wave intensity to temperature-attributable mortality, the activation of heat wave thresholds in prevention plans at a smaller geographical scale could lead to a reduction in attributable mortality.

**Authors**:

## Introduction

2025 has been the hottest year in Spain since records began, with a mean temperature that was 2.1 °C higher than the 1991–2020 average and 0.1 °C higher than the hottest summer to date, that of 2022 (AEMET, 2025). Despite this, the heat-wave attributable mortality figures reported by the Spanish mortality monitoring system (MoMo) of the Carlos III Institute of Health (MoMo 2025) show that in the period 1 June-30 September 2025, there were 3,824 deaths, whereas in the same period in 2022, a year that was not as hot, there were 4,732 deaths, i.e., 23.7% more attributable deaths than in 2025.

There are many factors that might account for the lower mortality in 2025, starting with the fact that regions which experience the highest temperatures may display greater or lesser adaptation to heat waves, as there is not only geographical heterogeneity in this respect (Follos et al. 2021) but also differing vulnerability (López-Bueno et al. 2022). Other factors that can have an influence include the point in time when heat waves occur (in that heat waves at the beginning of the hot months tend to have a greater mortality impact than those that occur later on), the duration of such heat waves and their number (Díaz et al. 2002). Furthermore, improvements, whether of a socio-economic nature (greater use of air conditioning or refrigeration systems), or in terms of health, urban infrastructures and building (thermal insulation and energy renovation), and even demographic distribution, can affect the health impact of heat waves (Navas-Martín et al., 2022), though in the years between 2022 and 2025 the variation in these variables can be considered to be of little significance at a national level (INE; 2025).

From a public health prevention standpoint, one factor which did change between 2022 and 2025 was the geographical resolution of the annual high temperature prevention plans implemented by the Spanish Ministry of Health. The 2022 prevention plan relied on determining the heat wave definition threshold temperature by reference to 52 provincial values (Minsan 2022a, b). This was achieved by using maximum daily temperature data sourced from a given observatory representative of each province along with daily mortality data for the province as a whole, so that Spain was divided into 52 regions. From 2024 onwards, however, this same High Temperature Prevention Plan (Minsan 2024) divided Spain into 182 regions with similar climatic behaviour (isoclimatic zone) and used data from all the observatories situated in each region, thus going from maximum daily temperature data drawn from 52 observatories to data sourced from more than 1,100 observatories, plus daily mortality corresponding to every town situated in each isoclimatic region (López-Bueno et al. 2024).

This study’s designated aim was thus to analyse whether this change in prevention plans from 2024 onwards could have influenced the decrease in heat-wave attributable mortality observed in 2025 with respect to 2022.

## Materials and methods

Since the data furnished by the MoMo system pertain only to the provincial level, it is to this level alone that this study refers (MoMo 2025).

As our dependent variable, we used attributable mortality in each province, as determined by MoMo for the period 1 June-30 September for the years between 2015, when this monitoring system first came into operation, and 2025.

As the independent variable, we used annual heat wave intensity (HWI) defined by reference to the maximum daily temperature registered by each provincial observatory (Tmax) and the heat wave definition threshold temperature (Tthreshold) determined for each province by the Ministry of Health in its prevention plans, using the methodology proposed by Díaz et al., (Díaz et al. 2006). From 2015 to 2023, we used the provincial threshold temperature (Tthreshold) for that period (Minsan, 2015), and from 2024 to 2025, we used the provincial Tthreshold for the relevant period (Minsan 2025). It should be noted that, although mortality was reported at a provincial level, in 2024 and 2025 public warnings and prevention measures were nevertheless implemented at the level of the 182 isoclimatic zones, with no mortality breakdown by isoclimatic region being available to date.

HWI is defined as follows:

$$\begin{array}{c}HWI\;\left({}^0C\right)\;=\;\underset{}{\sum\left(Tmax-Tthreshold\right)\;if\;Tmax\;>\;Tthreshold}\\0\;if\;Tmax\;<\;Tthreshold\end{array}$$

The summation extends to all heat wave days in that year.

**Fig. 1:**
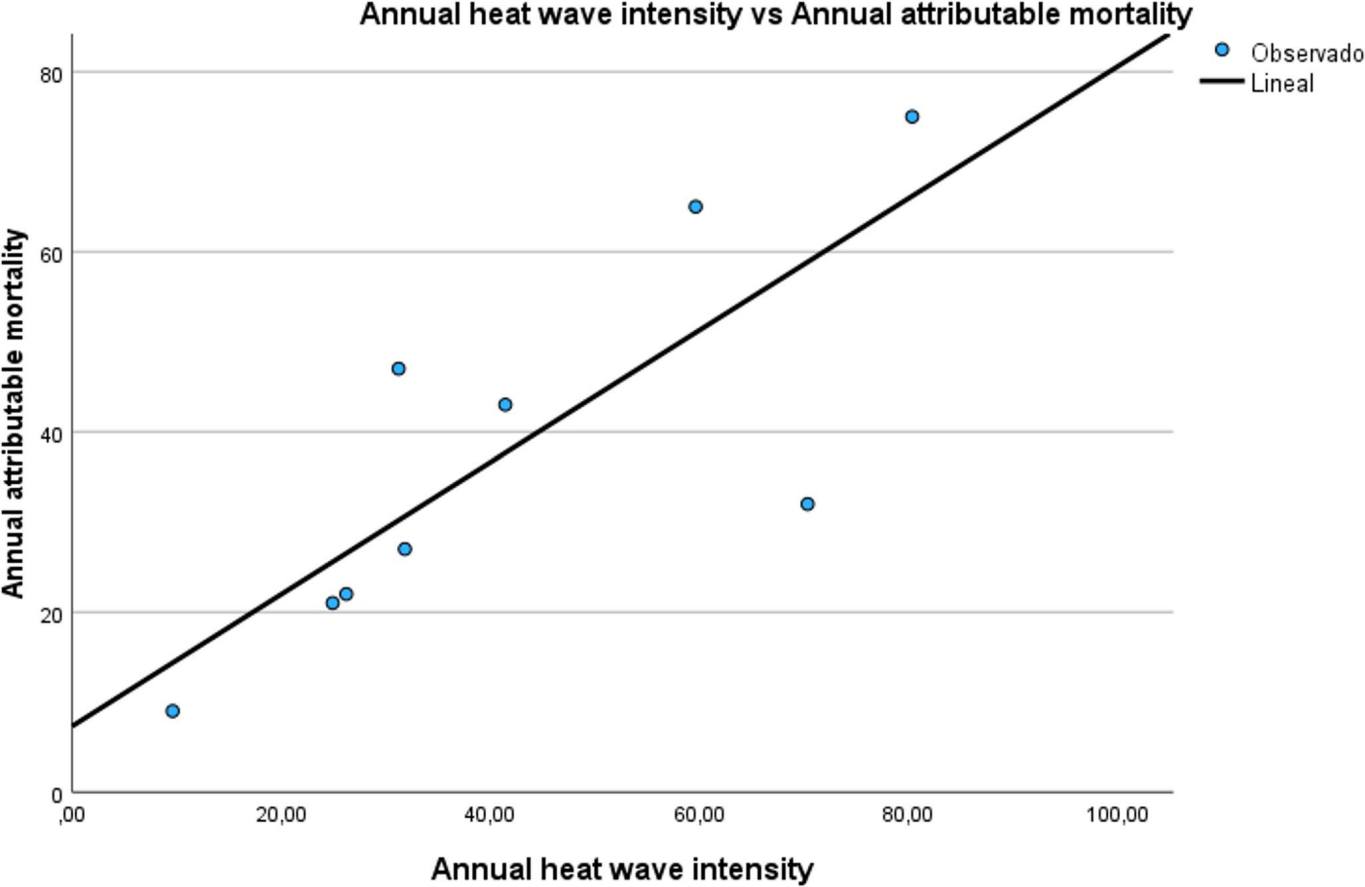
Linear adjustment between annual heat wave intensity and annual attributable mortality for Cordoba across the period 2015–2023

Based on the annual attributable mortality data for each province and its respective HWI, we first plotted a graph on which the X-axis corresponded to HWI and the Y-axis to mortality attributable to the heat waves recorded in that year, and then performed a linear adjustment. The slope of this linear adjustment (B) thus represented the increase in annual attributable mortality for every one-degree rise in HWI. This was done for each province, and for the period 2015–2023 versus 2024–2025. By way of example, Fig. 1 shows the linear adjustment for the province of Cordoba (Fig. 1).

## Results

Table 1 shows the results corresponding to the HWI values for each province across the period 2015–2025, the attributable mortality in each period, and the values of the slopes of the linear adjustments made for all Spanish provinces for the periods 2015–2023 and 2024–2025.

**Table 1:** Date by Province and for Spain overall: heat wave intensity across the period 2015–2025; attributable mortality across the period 2015–2025; slope of linear adjustment for the period 2015–2023; slope of linear adjustment for the period 2024–2025

Province	Heat wave intensity across the period (2015–2025) (ºC)	Attributable Mortality (2015–2025)	Slope (B) period (2015–2023) (deaths/ºC) (CI 95%)	Slope (B) period (2024–2025) (deaths/ºC)**
**A Coruña**	204.5	411	3.305* (1.088 5.522)	3.106
**Álava**	190.4	121	0.413* (0.107 0.719)	0.398
**Albacete**	417.5	303	0.277 (−0.188 0.742)	0.392
**Alicante**	442.4	1273	1.997* (0.854 3.140)	1.267
**Almería**	142.3	191	0.533* (0.021 1.045)	−0.260
**Asturias**	928.0	211	0.605 (−1.653 2.863)	0.158
**Ávila**	388.6	211	0.592* (0.455 0.729)	0.295
**Badajoz**	397.0	469	1.666* (0.704 2.628)	2.108
**Baleares (Isles)**	17.9	114	0.935 (−3.508 5.378)	−0.851
**Barcelona**	52.6	1013	22.486* (14.962 30.010)	12.488
**Bizkaia**	649.0	631	0.714* (0.432 1.016)	2.016
**Burgos**	155.2	191	1.518* (1.050 1.986)	0.649
**Cáceres**	211.0	288	1.117* (0.640 1.575)	1.729
**Cádiz**	126.7	236	−0.507 (−2.567 1.553)	0.000
**Cantabria**	51.4	76	1.177 (−0.948 3.302)	0.924
**Castellón**	288.1	411	0.997* (0.476 1.518)	1.885
**Ciudad Real**	235.8	399	1.339* (0.712 1.966)	1.867
**Córdoba**	375.3	341	0.733* (0.316 1.150)	0.359
**Cuenca**	651.3	309	0.443* (0.212 0.674)	−2.692
**Girona**	133.4	383	2.276* (1.696 2.856)	0.763
**Granada**	674.6	537	0.267 (−0.952 1.486)	−0.611
**Guadalajara**	75.6	89	0.609* (0.262 0.956)	0.256
**Guipúzcoa**	222.0	261	0.440 (−0.399 1.279)	7.692
**Huelva**	329.7	207	0.302 (−0.121 0.725)	1.047
**Huesca**	569.1	372	0.653* (0.484 0.822)	0.529
**Jaén**	716.1	666	0.658* (0.589 0.727)	0.702
**La Rioja**	190.5	183	0.732* (0.609 0.855)	−0.339
**Las Palmas G.C.**	80.0	44	0.000*	0.000
**León**	209.2	268	1.308* (1.018 1.598)	0.824
**Lleida**	474.2	266	0.448* (0.354 0.542)	0.158
**Lugo**	52.8	126	2.123* (1.702 2.544)	1.133
**Madrid**	897.3	4706	7.727* (3.832 11.622)	12.000
**Málaga**	33.0	268	5.202* (3.460 6.944)	3.936
**Murcia**	817.5	55	0.008 (−0.023 0.039)	0.848
**Navarra**	230.8	441	1.288* (1.607 0.969)	0.757
**Ourense**	274.3	177	0.627* (0.500 0.754)	1.838
**Palencia**	178.7	218	1.201* (0.987 1.415)	0.678
**Pontevedra**	366.7	582	1.202* (1.686 0.718)	0.488
**Salamanca**	437.7	434	0.994* (0.571 1.417)	0.663
**Sta Cruz Tenerife**	150.2	205	1.208* (0.814 1.602)	0.000
**Segovia**	178.2	104	0.791* (0.634 0.948)	−0.373
**Sevilla**	190.8	653	4.197* (2.917 5.477)	4.895
**Soria**	169.1	66	0.319* (0.288 0.350)	0.500
**Tarragona**	23.7	97	4.614* (1.831 7.397)	10.391
**Teruel**	236.5	68	0.324* (0.204 0.444)	1.379
**Toledo**	409.9	853	1.561* (1.073 2.049)	1.868
**Valladolid**	54.3	445	2.232* (0.145 4.319)	3.143
**Valencia**	106.1	853	1.220* (0.675 1.765)	1.257
**Zamora**	228.2	127	0.465* (0.326 0.604)	0.990
**Zaragoza**	426.9	778	1.589* (0.848 2.330)	−0.211
**Spain Overall**	284.5	435	1.738 (0.783 2.694)	1.660

*Significant slope *p* < 0.05

**The slope is significant at *p* < 0.001 and the standard error is zero (note that there are only two points)

In other words, according to the B values in each period, for every degree of annual heat wave intensity, mortality in Spain increased on average by 1.74 deaths/⁰C of HWI in the period 2015–2023 versus 1.66 deaths/⁰C of HWI in the period 2024–2025. This difference is not statistically significant according to Student’s t-test (*p* < 0.01).

Although these differences in B values may seem small when applied to calculating annual heat-attributable mortality, they nevertheless yield relevant results. In 2025 for instance, the HWI for Spain as a whole, obtained as the summation of the HWIs for each province, was 2,346 °C. The attributable mortality for this year, calculated on the basis of the values of the slope for the period 2015–2023, would amount to 4,082 attributable deaths, but if this same attributable mortality were calculated using the values of the slope corresponding to 2024–2025, it would come to a figure of 3,894, i.e., 188 fewer deaths. Of the difference of 908 attributable deaths between 2022 and 2025, a total of 188 (20.7%) could be linked to the improvement in heat-wave prevention plans at a geographical level. These 908 deaths are calculated as the difference between the 4,732 deaths attributable to heat waves in year 2022 according to MoMo (MoMo 2025) and the 3,824 deaths attributable in year 2025.

**Fig. 2:**
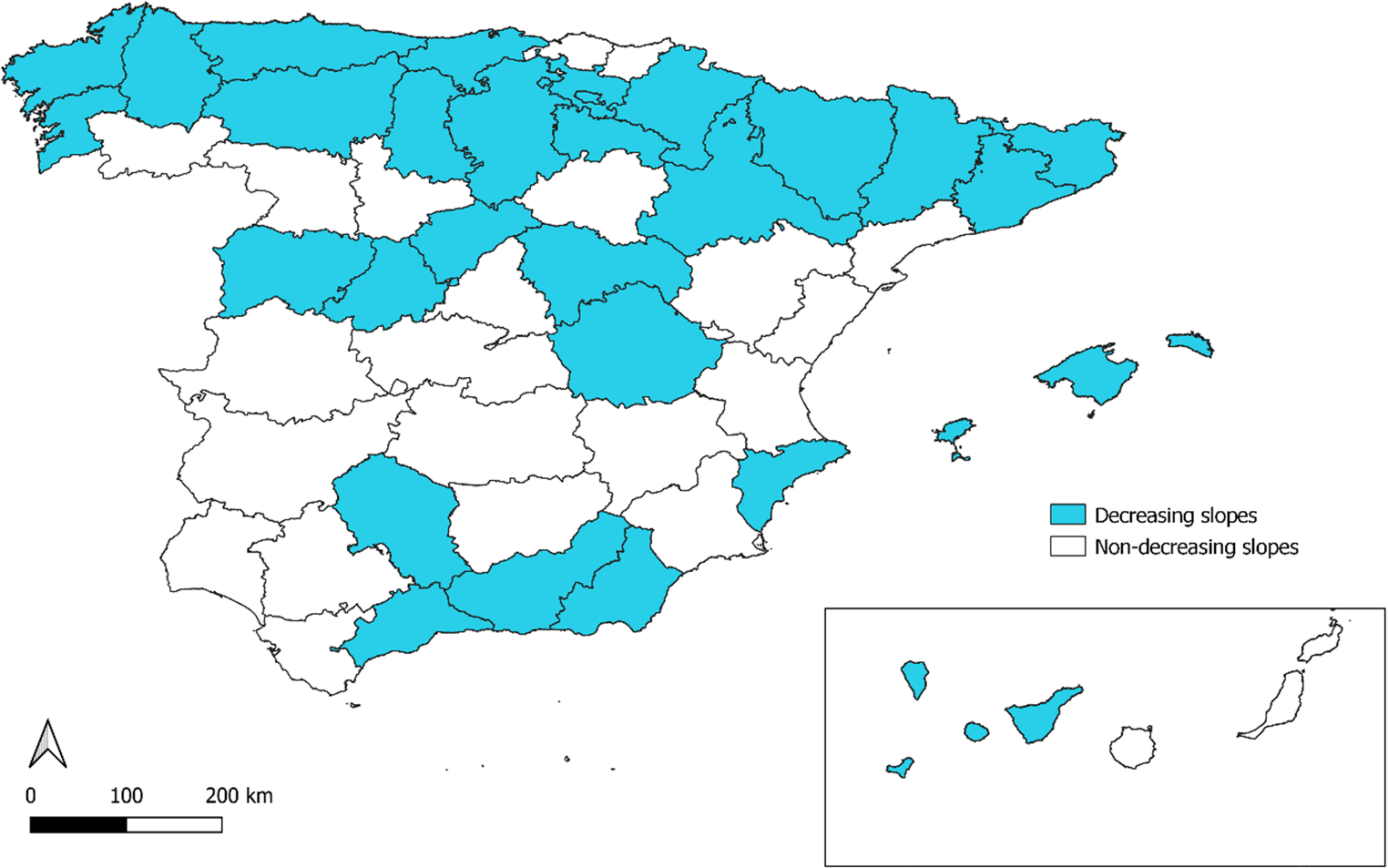
Geographical distribution of the provinces in which the slope of linear adjustment descended (shown in blue) in the period 2024–2025, as compared to 2015–2023. Shown in white, those provinces in which this slope ascended in the period 2024–2025, as compared to 2015–2023

Figure 2 shows the geographical distribution of the provinces in which the slope of linear adjustment descended across the period 2024–2025 with respect to 2015–2023. This analysis at a geographical level indicated that in 29 of Spain’s 50 provinces (58%), the impact of HWI on annual attributable mortality, as measured by the value of the slope of the line of fit (B), decreased, if its values for the period 2015–2022 are compared to those for 2024–2025. Its spatial distribution shows that, despite the high degree of heterogeneity, at a general level this was more visible in provinces in the north than in the south of the country, and above all, in coastal as opposed to inland provinces.

## Discussion

The decrease in heat-attributable mortality in 2024–2025 over 2015–2023 could be linked to the improvements made to public health prevention plans, assuming that the other variables mentioned in the Introduction as influencing heat-attributable mortality, have remained constant or undergone little change. These factors, such as income level and socioeconomic and demographic characteristics at the provincial level in Spain, have not changed significantly in the last two years, according to data from the National Institute of Statistics (INE, 2025).

The effectiveness of heat-wave prevention plans in reducing mortality in Spain has already been confirmed by the decrease in heat-attributable risks (ARs) observed after the implementation of prevention plans in 2004, with a reduction in AR per degree of threshold temperature values, from 14% before such plans were implemented (period 1983–2003) to just over 1% thereafter (period 2004–2013) (Díaz et al. 2018). The results of the geographical representation of the slopes at a provincial level indicate differentiated geographical patterns, one running north-south and the other, inland-coast. This behaviour pattern could be explained by reference to the results of two studies conducted in Spain on vulnerability to heat and population adaptation. These studies showed that in Spanish provinces in which heat waves were more frequent (inland areas and provinces in the south), vulnerability to heat was lower (López-Bueno et al. 2022) and the possibilities of adaptation greater than in those provinces that were less accustomed to heat waves (Navas-Martín, et al., 2022), with this effect being controlled for socio-economic factors, demographic factors and those related with urban infrastructures and building quality. Furthermore, the urban heat island effect described by Cuerdo-Vilches et al. (2023) shows that in coastal cities, the impact on heat wave morbidity and mortality is mainly associated with minimum temperatures (Tmin), whereas in cities lying in the interior, it is associated with maximum temperatures (Tmax), something that might serve to account for the differences observed between coastal and inland areas.

The results of this study may indicate that better determination of the maximum daily temperature to which the population is exposed, using a greater number of observatories that record the climatic characteristics of each area, could result in an improvement in the activation of heat-wave prevention plan thresholds and, by extension, a decrease in attributable mortality.

In addition, Spanish prevention plans are based on an “epidemiological” determination of the heat wave definition threshold temperature (Montero et al. 2012). This temperature is set by calculating the maximum daily temperature above which there is a statistically significant increase in population mortality. Using these temperatures enables the socio-economic, demographic, social and infrastructural characteristics of each place to be taken into account more effectively, and thus be more effective in prevention terms than are temperatures exclusively determined on the basis of the climatological characteristics of each place (Andersen et al. 2021).

Therefore, in order to implement heat wave prevention plans, it seems that using climatically homogeneous areas in terms of temperature may be more advisable than using purely administrative geographical divisions such as provinces.

In the case of Spain, this higher geographical resolution (182 isoclimatic regions compared to 52 provinces) entails an increase in the number of meteorological observatories where temperatures must be predicted. In the Spanish Prevention Plan, the number of meteorological observatories has increased from 52 (one representing each province) to more than 1,000 by using isoclimatic regions (all those existing in each region) (López-Bueno et al. 2024), which complicates the management of meteorological data. This is preliminary data management work to determine whether or not the threshold temperatures for defining a heatwave in each isoclimatic region are exceeded and to activate the corresponding public health measures set out in the action plan (Minsan 2024).

Another factor that may influence the comparison of mortality attributable to heat waves in 2022 and 2025 is the shift in mortality in relation to the previous winter’s flu epidemics. Some studies (Rocklöv et al., 2008) suggest that winter mortality in the previous year may influence lower mortality in the following summer, i.e., a shift in mortality due to a harvest effect. In other words, lower winter mortality from influenza would be associated with higher mortality from heat waves. The flu in the winter of 2022 was particularly severe, with nearly 10,000 deaths attributable to flu and influenza (Minsan 2022a, b), while in 2024–2025, mortality attributable to flu was 1,800 people (Redacción Médica 2025). Therefore, this lower mortality from influenza in 2025 compared to 2022 should have led to higher mortality in the summer of 2025 due to heat waves, which did not seem occur.

### Limitations

This study’s main limitation lies in the fact that in the latter period there were only two years of analysis, so that the values of the slopes obtained in this second period might not be wholly representative. Therefore, it is difficult to determine whether this represents a structural change or short-term variability that may not be sustained over time.

Furthermore, because it was an extremely hot year, 2025 enjoyed wide coverage by the mass media, which possibly led to higher awareness on the part of the population and the authorities involved in the management of health risks stemming from heat wave temperatures. Evidence of such mass media influence has been seen, for example, in the case of Odisha (India), where continuous exposure to messages disseminated by television and the press was associated with a significant reduction in heat wave mortality (Das 2016). There are also the limitations involved in using mortality attribution data based on a mortality monitoring system (MoMo) which may probably be underestimating the impact that heat wave temperatures have on mortality (Barceló and Saez 2025). Even so, on being a constant bias across the period, its effect would be controlled for throughout the series.

### Conclusion

The application of a high temperature prevention system based on the use of heat wave definition temperatures on a scale smaller than the provincial (isoclimatic regions) may have had an influence in reducing the impact that heat waves have had on attributable mortality in Spain, estimated at 20.7%. It is difficult to determine whether this represents a structural change or short-term variability that may not be sustained over time. That said, however, this study should be extended to a longer period in future, to prevent biases related with anomalous behaviours in any given year.

## Data Availability

The data used in this manuscript cannot be shared for reasons of confidentiality.

## Declarations

### Disclaimer

The views expressed by the authors are not necessarily those of the institutions with which they are affiliated.
